# Comparison of Transverse Cancellous Lag Screw and Ordinary Cannulated Screw Fixations in Treatment of Vertical Femoral Neck Fractures

**DOI:** 10.1111/os.12503

**Published:** 2019-07-23

**Authors:** Qiang Dong, Zhe Han, Yin‐Guang Zhang, Xiang Sun, Xin‐Long Ma

**Affiliations:** ^1^ Department of Traumatology Tianjin Hospital Tianjin China; ^2^ Department of Orthopaedics Tianjin Hospital Tianjin China

**Keywords:** Femoral head necrosis, Femoral neck shortening, Nonunion, Transverse cancellous lag screw, Vertical femoral neck fractures

## Abstract

**Objective:**

To compare the clinical therapeutic effect of transverse cancellous lag screw (TCLS) fixations and ordinary cannulated screw (OCS) fixations for vertical femoral neck fractures.

**Methods:**

A total of 62 eligible patients with an average age of 56.2 years (range, 19–45 years; 40 male and 22 female) with Pauwels’ type III femoral neck fractures were recruited in our study from January 2016 to December 2017. Among the patients, 30 underwent TCLS fixation (TCLS group), and the others were treated with OCS fixation (OCS group). The baseline data, perioperative outcomes (operative time, intra‐operative blood loss, reduction quality, and hospital time), postoperative outcomes evaluated by a variety of scales including visual analogue scale (VAS) score, EuroQol five dimensions questionnaire (EQ‐5D) and Harris hip scores (HHS), and complications (nonunion, femoral head necrosis, femoral neck shortening, and failure of fixation) of the two groups were recorded to compare at 12‐month follow‐up.

**Results:**

The mean follow‐up time of included patients was 13.4 ± 1.6 months in the TCLS group and 13.7 ± 0.9 months in the OCS group. There was no statistically significant difference in the baseline data as well as perioperative outcomes, including operative time, intra‐operative blood loss, the hemoglobin difference before and after treatment, quality of reduction, and hospital time between two groups. Likewise, the VAS score, the EQ‐5D score, and complications rates including nonunion and femoral head necrosis had no distinct difference in two groups. However, HHS in the TCLS group were superior to those in the OCS group at 12‐month follow‐up, and the femoral neck shortening rate was prominently reduced in the TCLS group when compared with the OCS group.

**Conclusions:**

Treating vertical femoral neck fractures with the TCLS technique could significantly improve hip functional recovery and reduce the postoperative femoral neck shortening rate. The present study provides novel insight for the treatment of vertical femoral neck fractures.

## Introduction

Femoral neck fractures refer to the fractures occurring between the femoral head and the basal part of the femoral neck. They are caused by a variety of reasons and are among the most common types of hip fractures^1^. Femoral neck fractures often occur in the elderly. Their incidence is increasing with longer life expectancy, especially with the aging population, and they have become a serious social problem^2^. Recent epidemiological surveys show that femoral neck fractures accounted for approximately 3.8% of fractures in adults, 50% of which occurred in the older population^3^, ^4^. Osteoporosis is one of the most important factors leading to femoral neck fractures in the elderly,^5^ and is caused by reduced activity and decreased levels of various hormones^6^, ^7^. Most femoral neck fractures in younger individuals with normal bone are due to high‐energy trauma such as sports injuries, car accidents, and falling from heights^8^.

Pauwels’ classification has been widely used to evaluate the type and severity of femoral neck fractures, especially in young people, since first recognized in the 1930s^9^. Femoral neck fractures were divided into three types on the basis of their degree of verticality: type I, less than 30 degrees; type II, 30–50 degrees; and type III, >50°^10^. Vertical femoral neck fractures (Pauwels’ type III) in young adults, caused by a large axial force across the hip, are associated with increased risks of fixation failure, malunion, nonunion, and osteonecrosis^11^, ^12^. Despite great advances in fixation implants, there still exists debate on the appropriate therapeutic schedule for these injuries^13^.

Given the minor trauma and its reliable fixation effect, closed reduction and internal fixation with multiple cannulated screws has become the most common clinical application for the treatment of femoral neck fractures^14^. Despite there being no obvious difference in the vertical load resistance between two‐cannulated‐screw and three‐cannulated‐screw fixation from a biomechanic prospective^15^, ^16^, most doctors believe that three cannulated screws have a better effect in controlling impaction during weight‐bearing to stabilize the fracture and facilitate healing^17^, ^18^. The optimal construct for femoral neck fixation was an inverted triangle configuration with three parallel screws, which had outstanding effects in regard to anti‐twisting and anti‐tensility. However, for vertical femoral neck fractures, with the treatment of three cannulated screw fixation, the frequency of complications such as nonunion and femoral head necrosis incidence was still high^11^. Studies have revealed several alternative means of fixation for femoral neck fractures using three cannulated screws^19^, ^20^, ^21^. Unfortunately, this uncommon natural fracture type has been reported in only a few studies, which are retrospective and non‐comparative, so their conclusions are limited.

Based on previous studies and our experience, we hypothesized that transverse cancellous lag screw (TCLS) configuration was superior to ordinary cannulated screw (OCS) configuration in the treatment of Pauwels’ type III femoral neck fractures, with better functional recovery and few complications. Therefore, the main goals of this study are to: (i) propose a novel method of screw configuration for treatment of vertical femoral neck fractures; (ii) explore the therapeutic effect of the TCLS technique compared with the OCS technique; and (iii) summarize the existing limitations and the possible direction for further research.

## Materials and Methods

### *The Inclusion and Exclusion Criteria*

Inclusion criteria: (i) patients aged 18–65 years old; (ii) displaced transcervical femoral neck fracture with vertical orientation (Pauwels’ angle>50 degrees); (iii) no history of hip disease; (iv) unilateral femoral neck fracture; and (v) postoperative follow‐up time of more than 1 year.

Exclusion criteria: (i) surgical area with poor skin conditions or skin diseases; (ii) severe osteoporosis; (iii) pathological fractures or old fractures (more than 14 days); (iv) comorbid severe cardiovascular and cerebrovascular diseases, mental illness, and abnormal liver and kidney function; (v) pregnant or lactating women; (vi) autoimmune diseases; (vii) blood disorders; and (viii) incomplete clinical data.

### *Included Patients*

**Figure 1:**
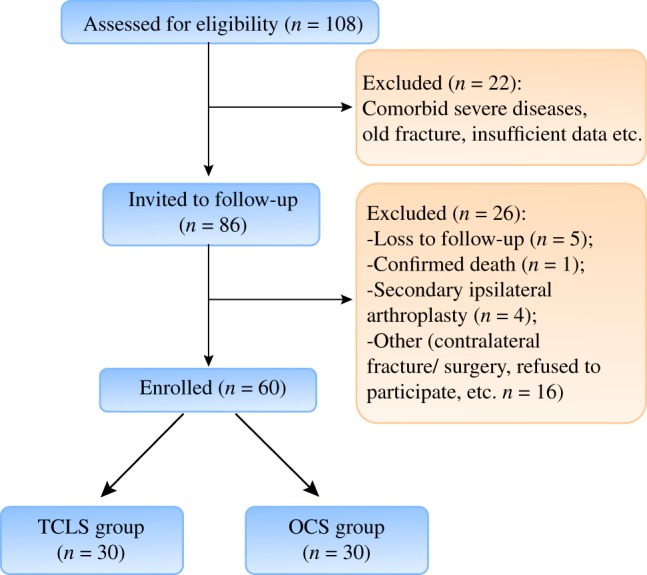
Flowchart of patients in both groups.

One hundred and eight patients with fresh Pauwels’ type III femoral neck fractures were recruited from January 2016 to December 2017 in Tianjin Hospital. Of these, 22 patients did not fulfill the inclusion criteria, 15 patients were lost to follow‐up,4 patients had a Secondary ipsilateral arthroplasty and 5 patients withdrew from this study for other reasons. (Fig. 1). The demographic characteristics and clinical profiles including age, gender, American Society of Anesthesiologists (ASA) score, history of smoking and drinking, injury mechanism, fracture side, time to surgery, and follow‐up time of patients were recorded. Among 62 patients, 30 underwent TCLS fixation (TCLS group), and the others were treated with ordinary cannulated screw fixation (OCS group). All participants obtained informed consent, and the current study was approved by the ethics committee of Tianjin Hospital.

### *Fracture Assessment on Plain Radiographs*

Preoperative anteroposterior position (AP) plain radiographs of the hip were performed to confirm whether the patient suffered from a vertical femoral neck fracture. If rotation of the lower limb precluded accurate measurement of the coronal fracture angle on the preoperative radiographs, this was noted and immediate post‐fixation radiographs were used. The vertical fracture angle on these radiographs was determined by comparing a best fit of the fracture line with a line horizontal (perpendicular) to the femoral shaft as previous described^10^.

### *Surgical Procedure*

#### *Anesthesia and Surgical Position*

The patients were placed in a supine position with the affected side's hip elevated 15–20 cm under continuous epidural anesthesia. The unaffected side lower limb was maintained in abduction as soon as possible and kept the bend one's knees and coxa. Meanwhile, the affected side lower limb was fixed at 30° internal rotation and 30° abduction. Then, the fracture was treated with preoperative traction reduction under the guidance of C‐arm fluoroscopy.

#### *Approach and Exposure*

After obtaining satisfactory reduction, inferior 5‐cm longitudinal incision of the femoral trochanter was performed, cutting and separating subcutaneous tissue, exploring the lateral bone cortex. In the OCS group, the first k‐wire was inserted parallel into the femoral head across to the femoral neck. Under the intraoperative X‐ray control, the other two k‐wires were inserted to make an inverted triangle. In the TCLS group, the anterior two k‐wires were placed exactly as they were in the OCS group, which were inserted at a 125° angle from the femoral shaft. The third k‐wire was placed posterior, inserted from the lateral aspect of the greater trochanter towards the posterior, which was intersected with the fracture line at a 90° angle. It should be advanced to within 5 mm of the subchondral bone to meet the requirements of the tip apex distance.

#### *Screw‐Setting*

Appropriate partial threaded three cannulated screws or cancellous lag screws were twisted until the screws were tight. The wound was sutured step by step when the k‐wires were pulled out.

### *Postoperative Management*

According to the actual situation of the patient, early functional exercise is carried out under the guidance of the physiatrician. The patients were given oxygen, electrocardiogram monitoring, nutritional support, and adequate antibiotics to prevent infection postoperatively. AP plain radiographs and CT of the hip were reexamined until the patients were in a stable condition. Individual postoperative rehabilitation plans were formulated by experienced physiatricians. In general, the patients were ambulated with axillary crutches and touch weight bearing for 6 weeks, and partial weight bearing for another 6 weeks. Each patient was followed up clinically and radiologically at 3, 6, and 12 months postoperatively, to scientifically assess quality of life, hip joint function, and complications.

### *Data Collection and Outcome Evaluation*

The raw observation data for the two groups were recorded and compared as follows.

#### *Operative Time*

The time of surgery was recorded from the beginning of skin incision until surgical closure, which could reflect the proficiency of the operators for these two different techniques and risk of infection.

#### *Blood Loss*

Intraoperative blood loss was measured by summation of the hemorrhage through the suction instruction and the bleeding volume at the gauzes. Meanwhile, the concealed hemorrhage was calculated by the difference in value of the hemoglobin before and after surgery using routine blood examination.

#### *Quality of Reduction*

As previously described by Haidukewych *et al*.^15^, the quality of fracture reduction was graded as excellent (<2 mm of displacement and <5° of angulation in any plane), good (2 to 5 mm of displacement and/or 5 to 10° of angulation), fair (>5 to 10 mm of displacement and/or > 10° to 20° of angulation), or poor (>10 mm of displacement and/or > 20° of angulation, or any varus).

#### *Hospital Time*

The inpatient days were counted from the time of emergency admission to the time of discharge on doctor's orders. Following a standardized process, all patients underwent corresponding surgery within 72 h. Hence, the hospital time represented perioperative recovery and expenditure of patients in the two groups.

#### *Visual Analogue Scale*

The visual analogue scale scoring system was performed to estimate the pain level of the patients. The VAS pain scoring standard (scores from 0 to 10) was as follows: 0 = painless; less than 3 = mild pain that the patient could endure; 4–6 = patient was in pain that could be endured and was able to sleep; and 7–10 = patient had intense pain and was unable to tolerate the pain.

#### *EuroQol Five Dimensions Questionnaire*

The EuroQol five dimensions questionnaire (EQ‐5D) was a patient‐reported instrument used to measure living quality, which was developed by the EuroQol group^22^. The questionnaire includes five questions concerning five different dimensions: mobility, self‐care, usual activities, pain/discomfort, and anxiety/depression. The specific value set for Chinese patients using the EQ‐5D questionnaire was drawn from the study of Luo *et al*.^23^

#### *Harris Hip Score*

The Harris hip score (HHS, which was first published in 1969) was used to evaluate the postoperative recovery of hip function in an adult population^24^. The HHS score system mainly includes four aspects: pain, function, absence of deformity, and range of motion. The score standard had a maximum of 100 points (best possible outcome) and included pain (one item, 0–44 points), function (seven items, 0–47 points), absence of deformity (one item, four points), and range of motion (two items, five points).

#### *Complications*

Clinical and radiographic detections were performed to estimate the fracture healing, and femur head necrosis, femoral neck shortening and fixation failure during follow‐up. Femoral head necrosis was evaluated radiographically according to Ficat criteria^25^. Union of the fracture was defined as fracture line having completely disappeared and bone trabecular structure as basically consistent with that of normal people, whereas nonunion was construed as persistence of the fracture line 6 months after the surgical procedure^19^. Femoral neck shortening was measured as described by Zielinski *et al*.^26^, and was assessed in the horizontal (abductor moment arm shortening) and vertical plane (femur length decrease). The type of internal fixation failure included screw out, screw loosening, and screw extraction, which were surveyed at each imaging examination.

### *Statistical Analysis*

All statistical analyses were performed using SPSS 21.0 software (IBM Software, Chicago, IL, USA) and GraphPad (vision 7.0, USA). For categorical variables, the χ^2^‐test and Fisher's exact test were used. For quantitative variables, the data were expressed as mean ± SD, and compared by using the Student's *t*‐test or ANOVA between two groups. A value of *P* < 0.05 indicated a statistically significant difference.

## Results

### *General Results*

A total of 62 patients, 40 men and 22 women, with an average age of 56.2 years (range, 19–65 years), were recruited for our research. The general characteristics of enrolled patients are described in Table 1 and a flowchart of the study is displayed in Fig. 1. Overall, 30 patients were treated with TCLS fixation and others were treated with OCS fixation, and there were no statistically significant differences in age (55.7 ± 7.9 years *vs* 56.3 ± 8.2 years, *P* = 0.770), gender (male/female, 18/12 *vs* 22/10, *P* = 0.472), ASA score higher than 2 (10.0% *vs* 6.3%, *P* = 0.588), smoking (23.3% *vs* 15.6%, *P* = 0.443) and drinking history (20.0% *vs* 18.8%, *P* = 0.901), injury mechanism (high‐energy trauma, 66.7% *vs* 59.4%; low‐energy trauma, 33.3% *vs* 40.6%, *P* = 0.553), fracture side (left/right, 14/19 *vs* 16/13, *P* = 0.316), time to surgery (3.2 ± 0.8 days *vs* 3.4 ± 0.7 days, *P* = 0.298), and follow‐up time (13.4 ± 1.6 months *vs* 13.7 ± 0.9 months, *P* = 0.363).

**Table 1:** Comparison of baseline data between the two groups

Variables	Groups		*P‐*value
	TCLS	OCS	
Age (years)	55.7 ± 7.9	56.3 ± 8.2	0.770
Gender (%)			
Male	18 (60.0)	22 (68.8)	0.472
Female	12 (40.0)	10 (31.3)	
ASA score >2 (%)	3 (10.0)	2 (6.3)	0.588
Smoking (%)	7 (23.3)	5 (15.6)	0.443
History of drinking (%)	6 (20.0)	6 (18.8)	0.901
Injury mechanism (%)			
High‐energy trauma	20 (66.7)	19 (59.4)	0.553
Low‐energy trauma	10 (33.3)	13 (40.6)	
Fracture side			
Left	14 (46.7)	19 (59.4)	0.316
Right	16 (53.3)	13 (40.6)	
Time to surgery (days)	3.2 ± 0.8	3.4 ± 0.7	0.298
Mean follow‐up time (months)	13.4 ± 1.6	13.7 ± 0.9	0.363

ASA, American Society of Anesthesiologists; OCS, ordinary cannulated screw; TCLS, transverse cancellous lag screw

### *Perioperative Outcomes*

All operations were performed successfully by the lead author (QD) for 48 patients (77.4%), a consultant orthopaedic surgeon for 10 patients (16.1%), and two junior surgeons for 4 patients (6.5%)(Table 2).

**Table 2:** Comparison of perioperative outcomes between the two groups

	Groups		
Variables	TCLS	OCS	*P‐*value
Operative time (min)	46.2 ± 20.9	40.9 ± 18.3	0.292
Intra‐operative blood loss (ml)	68.6 ± 25.3	62.4 ± 32.5	0.407
The hemoglobin difference before and after treatment(g/L)	2.5 ± 0.5	2.3 ± 0.6	0.161
The quality of reduction (%)			0.516
Excellent	14 (46.7)	13 (40.6)	
Good	8 (26.7)	12 (37.5)	
Fair	6 (20.0)	6 (18.8)	
Poor	2 (6.6)	1 (3.1)	
Hospital time(day)	7.1 ± 2.3	7.3 ± 1.4	0.678

OCS, ordinary cannulated screw; TCLS, transverse cancellous lag screw.

#### *Operative Time*

The mean operative time was 46.2 ± 20.9 h in the TCLS group and 40.9 ± 18.3 h in the OCS group. There was no significant difference between the two groups (*P* = 0.292). Our results suggest that this new technique would not prolong the operation time and increase risk of infection.

#### *Blood Loss*

The mean intraoperative blood loss was 68.6 ± 25.3 mL in the TCLS group, and 62.4 ± 32.5 mL in OCS group. There were no conspicuous differences between the two techniques (*P* = 0.407). At the same time, the hemoglobin difference before and after treatment was 2.5 ± 0.5 g/L in the TCLS group and 2.3 ± 0.6 g/L in the OCS group, revealing no evident distinction in the two groups (*P* = 0.161). Therefore, the TCLS technique would not increase blood loss when compared with the traditional technique.

#### *Quality of Reduction*

**Figure 2:**
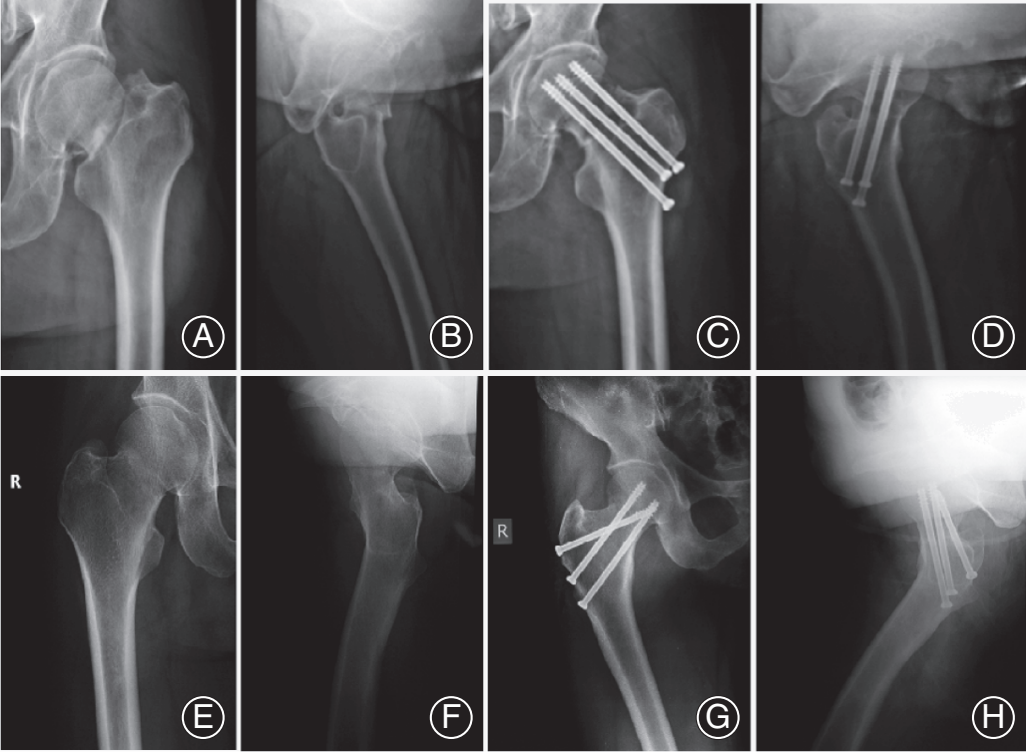
Different internal fixation techniques in two groups. (A, B) Preoperative and (C, D) postoperative radiographs of a 52‐year‐old man with Pauwels’ type III femoral neck fractures fixed with ordinary cannulated screw (OCS) technique; (E, F) Preoperative and (G, H) postoperative radiographs of a 38‐year‐old man with Pauwels’ type III femoral neck fractures fixed with the transverse cancellous lag screw (TCLS) technique.

Similarly, 27 patients had an anatomic reduction, 20 patients had a reduction in acceptable with minor concerns, 12 patients had a reduction in acceptable borderline, and 3 patients had a poor reduction, with no dramatic difference between TCLS and OCS groups (Table 2, Fig. 2,* P* = 0.516).

#### *Hospital Time*

The mean hospital time was 7.1 ± 2.3 days for the TCLS group and 7.3 ± 1.4 days for the OCS group, with no remarkable differences (*P* = 0.678). Our results indicated that usage of the TCLS technique for vertical femoral neck fractures would not impact perioperative recovery and increase expenditure for patients with vertical femoral neck fractures.

### *Postoperative Outcomes*

As shown in Table 3, functional outcomes in both groups had improved significantly at the postoperative follow‐up.

**Table 3:** Comparison of function outcomes between the two groups (mean ± SD)

	Groups		
Variables	TCLS	OCS	*P*‐value
VAS			
Pre‐FX VAS	1.7 ± 1.1	1.6 ± 1.3	0.746
3‐month follow‐up	2.8 ± 1.3	2.7 ± 1.4	0.772
6‐month follow‐up	2.6 ± 1.4	2.8 ± 1.3	0.562
12‐month follow‐up	2.2 ± 1.6	2.5 ± 1.5	0.450
EQ‐5D			
Pre‐FX EQ‐5D	0.85 ± 0.12	0.83 ± 0.15	0.566
3‐month follow‐up	0.43 ± 0.19	0.35 ± 0.25	0.163
6‐month follow‐up	0.68 ± 0.21	0.60 ± 0.24	0.169
12‐month follow‐up	0.71 ± 0.23	0.65 ± 0.20	0.277
HHS			
Pre‐FX HHS	91.2 ± 4.8	90.7 ± 5.2	0.696
3‐month follow‐up	64.5 ± 16.1	57.1 ± 18.6	0.100
6‐month follow‐up	74.3 ± 17.6	66.8 ± 18.2	0.105
12‐month follow‐up	81.5 ± 13.4	72.9 ± 17.7	0.036

EQ‐5D, EuroQol five dimensions questionnaire; FX, fracture; HHS, Harris hip score; OCS, ordinary cannulated screw; TCLS, transverse cancellous lag screw; VAS, visual analogue scale.

#### *Visual Analogue Scale*

The scores of VAS were 2.8 ± 1.3, 2.6 ± 1.4, and 2.2 ± 1.6 in the TCLS group at 3‐month, 6‐month, and 12‐month follow‐up, respectively, while these were 2.7 ± 1.4, 2.8 ± 1.3, and 2.5 ± 1.5 in OCS group at corresponding time points. It was shown that there was no statistical difference in post operative pain between the two techniques (*P* = 0.772 for 3 months, *P* = 0.562 for 6 months, and *P =* 0.450 for 12 months).

#### *EuroQol Five Dimensions Questionnaire*

The EQ‐5D method was used to access the positive life quality for the two groups. Our results showed that it was 0.43 ± 0.19, 0.68 ± 0.21, and 0.71 ± 0.23 in the TCLS group at 3‐month, 6‐month, and 12‐month follow‐up, respectively. For the OCS group, it was 0.35 ± 0.25, 0.60 ± 0.24, and 0.65 ± 0.20 in the TCLS group at 3‐month, 6‐month, and 12‐month follow‐up, respectively. To sum up, our results revealed that postoperative quality of life had no striking difference for the two techniques used in the treatment for vertical femoral neck fractures (*P* = 0.163 for 3 months, *P* = 0.169 for 6 months, and *P =* 0.277 for 12 months).

#### *Harris Hip Score*

The HHS appraisal system was performed to measure the postoperative hip function between the two techniques. The HHS score was 64.5 ± 16.1 and 74.3 ± 17.6 in the TCLS group, and was 57.1 ± 18.6 and 66.8 ± 18.2 in the OCS group at 3‐month and 6‐month follow‐up, respectively. There was no marked distinction in the postoperative hip functional recovery between the two groups over 6 months (*P* = 0.100 for 3 months and *P* = 0.105 for 6 months). However, the HHS score was 81.5 ± 13.4 in the TCLS group at 12‐month follow up, and was 72.9 ± 17.7 in the OCS group, suggesting that TCLS fixation was more beneficial to postoperative hip functional recovery (*P* < 0.05).

### *Complications*

**Figure 3:**
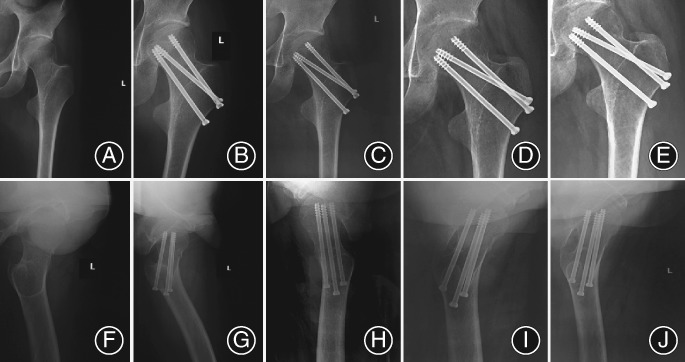
Radiograph showing fracture healing process in a 55‐year‐old woman fixed with the transverse cancellous lag screw (TCLS) technique. (A–E) The anterioposterior X‐ray review of proximal femur at 1 day, 3 months, 6 months, and 12 months after the operation. (F–J) The lateral X‐ray review of the proximal femur at 1 day, 3 months, 6 months, and 12 months after the operation.

After 12 months of follow‐up, bone union was achieved in 28 patients from the TCLS group (Fig. 3) and 29 patients from the OCS group (*P* = 0.696). At the same time, the femoral head necrosis was observed in 1 patient in the TCLS group and 3 in the OCS group (*P* = 0.333, Table 4). The femoral neck shortening was recorded among the patients with fracture healing, and our results showed that horizontal shortening (>5 mm) occurred in 26.7% of patients in the TCLS group, and significantly more in the OCS group (53.1%, *P* < 0.05). Furthermore, vertical femur shortening was more likely in the OCS group (50.0%) than the TCLS group (23.3%, *P* < 0.05). In addition, there were no statistically significant differences in the fixation failure rate between patients treated with these two techniques (0.0% *vs* 3.1%, *P* = 0.329). Taken together, we determined that the usage of TCLS configuration to treat vertical femoral neck fractures was better to avoid shortening of the femoral neck after fracture healing, without higher femoral head necrosis rate and fixation failure rate.

**Table 4:** Comparison of postoperative complications between the two groups

	Groups		
Variables	TCLS	OCS	*P*‐value
Nonunion/malunion (%)	2 (6.7)	3 (9.4)	0.696
Femur head necrosis (%)	1 (3.3)	3 (9.4)	0.333
Femoral neck shortening	8 (26.7)	17 (53.1)	0.034
(>5 mm, horizontal plane) (%)			
Femoral neck shortening	7 (23.3)	16 (50.0)	0.030
(>5 mm, vertical plane) (%)			
Failure of fixation (%)	0 (0.0)	1 (3.1)	0.329

OCS, ordinary cannulated screw; TCLS, transverse cancellous lag screw.

## Discussion

The incidence of femoral neck fractures has increased significantly in young and middle‐aged people, with the rise in high‐energy traumas such as traffic injuries and high falling injuries^27^. Due to the special anatomy of the femoral neck and femoral head, the rate of nonunion and femoral head necrosis after femoral neck fracture is high^11^. Current studies recognize that early anatomic reduction, stable internal fixation, and protection of femoral head blood supply are the key points for successful surgery^15^. However, vertical femoral neck fractures (Pauwels’ type III) are identified as a more challenging injury, which require more rigid fixation to achieve uneventful fracture healing compared with low angle fractures^28^. Therefore, strategies for optimizing fixation stability in patients with vertical femoral neck fractures are still controversial.

Multiple cannulated screw fixation is a widely accepted technique for femoral neck fractures because it can reduce soft tissue damage enormously, it protects the biological environment, and it supports the biomechanical stability of fractures^18^, ^29^. In Pauwels’ classification, the shear force at the fracture end becomes greater with the increase in the number of fracture line clamp angles, which can also cause fracture instability^30^. Increasing evidence has revealed that there is a high incidence of complications in Pauwels’ type III femoral neck fracture patients fixed with three parallel hollow lag screws, such as nonunion and femoral head necrosis^31^, ^32^. Based on cannulated screw fixation, a good deal of research has attempted to explore the ideal number, position, and configuration of screws and hybrid construct methods for optimal fixation of this problematic fracture type through biomechanical study, finite element analysis, and clinical practice. For instance, using biomechanical analysis, Kuan *et al*. indicated that the addition of a cerclage wire to inverted triangle triple parallel hollow lag screws provided a more stable mechanical structure for vertical femoral neck fractures in comparison to the traditional internal fixation method^33^. Bishop *et al*. declared that addition of washers to cannulated screw fixation provided an improved compression force for the femoral neck, increased the maximum insertion torque of screws, and prevented the screw heads from penetrating the lateral cortex^34^. The usage of crossed divergent lag screws in femoral neck fracture fixation is a popular strategy, which was first reported on by Filipov *et al*.^35^ Theoretically, using non‐parallel distributed cannulated screws might lead to a substantial improvement in mechanical performance in proximal femurs and significantly enhance anti‐rotation and shear resistance to fracture ends, consequently allowing for controlled fracture impaction^35^.

Based on the above assumptions, a battery of biomechanical tests were performed to verify the structural stability of the proximal femur, which was fixed using the cross screw configuration method. Freitas *et al*. first proposed that usage of non‐parallel cannulated screws partly eliminated the shear strength existing in vertical femoral neck fractures in comparison to inverted triangle fixation^36^. Furthermore, crossed divergent lag screws for isolated vertical femoral neck fractures seemed to have similar biomechanical stability to fixed angle cephalon medullary intramedullary or femoral neck locking plates^28^. Inspiringly, some other studies have advocated that transverse lag screw construct was stiffer and stronger than sliding hip screws or parallel lag screws^19^. In contrast, Johnson *et al*. claimed that hip screw with titanium derotation screw fixation was superior to both modified cross screw configuration and traditional inverted triangle fixation in Pauwels’ type III fracture model, where the latter two groups also had no significant difference in establishing biomechanical stability^20^.

After refining previous studies of crossed divergent lag screw techniques, we put forward a novel way to place transverse lag screws, inserted them from the lateral aspect of the greater trochanter towards the posterior, which was intersected with the fracture line at a 90° angle. The improvement seen in structural stability with the TCLS technique might be explained as follows. First, this construction could not only improve the compression pressure and offer a multiplane structure to lock the fracture ends, but also was able to compress fracture ends dynamically during the healing progress. Second, a transverse screw placed through the fracture line at a 90° angle might achieve optimum control of shear forces in fracture ends, which is consistent with the previous findings^19^. Finally, there was the relative weak area in the proximal femur called Ward's triangle, which also lead to inconsistent and incomplete of structure of the femoral neck. Consequentially, a posterior transverse screw could transfer the bending moments from the femoral head and neck to the enhanced posterior lateral cortical support, which harmonized the function of the calcar femorale. For the above reasons, we speculated that the TCLS technique could provide a stable biomechanical environment for vertical femoral neck fractures, leading to endosteal healing and sprouting angiogenesis. To further confirm our hypothesis, we observed whether the TCLS technique was superior to the OCS technique in Pauwels’ type III fractures by systemically recording perioperative outcomes, functional outcomes, and complications at 12‐month follow‐up.

In the present study, we found that there were no statistically significant differences in terms of perioperative outcomes, EQ‐5D score, visual pain score, and complications including nonunion, femur head necrosis and failure of fixation in the two groups at 12‐month follow‐up. These results certainly indicated that these two techniques both achieved good results in the treatment of Pauwels’ type III femoral neck fractures. However, our data showed that there were advantages in using the TCLS technique over the OCS technique in postoperative HHS scores. In other words, the patients who received TCLS treatment had better recovery of hip function. Hence, it is worth noting that vertical fractures of the femoral neck might lead to poor hip function with traditional therapy and related comorbidities. In addition, femoral neck shortening, with an incidence of around 41.7% in all patients, was also a common complication after internal fixation of femoral neck fractures, which is believed to be related to bone resorption during the fracture healing^37^. In fact, we also realized that the majority of these fractures were comminuted. Therefore, our aim of achieving a good anatomic closed reduction was more difficult, resulting in femoral neck shortening, through the usage of multiple compression screws to improve fixed stability and to help control the shear forces. This phenomenon was also reflected in our observations. It was shown that both horizontal and vertical planes of femoral neck shortening were occurring in patients at 1 year postoperatively. It was also found that the TCLS technique provided better resistance to neck shortening than the OCS technique. This means that the usage of a rear transverse screw could be expected to neutralize shearing forces and effectively act as a buttress against varus malformation^38^.

Recently, the importance of crossed lag screw fixation in the treatment of Pauwels’ type III femoral neck fractures and its capacity to achieve acceptable outcomes had been demonstrated in several studies^19^, ^35^. Based on in‐depth biomechanical research, it was reported that a transverse screw could create an inferior buttress which resisted displacement of femoral neck fractures and achieve optimum compression and better control of shear forces^39^. Hawk *et al* 38. and Gumustas *et al* 32. also expounded the stability benefits for unstable femoral neck fractures by using a transverse screw in the calcar in addition to two cannulated screws parallel to the neck. However, Johnson *et al*. suggest the use of dynamic hip screws for Pauwels’ type III femoral neck fracture fixation, in view of the better biomechanical performance in synthetic femoral models than crossed cannulated screw configuration^20^. Parker et al. reported that transverse screws applied for fixation of femoral neck fractures were unconvinced, which accompanied with more nonunion and of femoral head necrosis^40^. Nevertheless, the study did not perform subgroup analysis based on gender, age, and bone quality, so its results were mixed with a number of confounding factors, and it did not reflect the real situation. In fact, more studies have reported that the addition of a transverse screw could improve fixation stability after anatomical reduction in younger patients with femoral neck fractures^32^, ^41^.

The limitations of this study are as follows. First, due to the small sample size and shorter follow‐up time, there was insufficient evidence to confirm the role of TCLS in the treatment of vertical femoral neck fractures. Second, there was an absence of control groups of other types of internal fixation such as intramedullary nail or dynamic hip screw, which makes it difficult to more precisely summarize improvement or defects in this new technique, and obstruct its clinical application on account of contrasting data. Finally, the observational indicators in the present study were not comprehensive enough: for example, more accurate quality of life assessments and mental evaluations could be adopted.

### *Conclusion*

Currently, the “gold standard” for treatment of vertical femoral neck fractures in the non‐elderly is still unclear and each patient should be given individualized treatment, taking into consideration the complex injury mechanisms. The present study suggests that using the TCLS technique to treat vertical femoral neck fractures could improve hip functional outcomes and reduce the rates of neck shortening. The present study might provide novel insight for treatment of Pauwels’ type III femoral neck fractures. However, future larger randomized trials comparing this novel technique with other fixation techniques is necessary to determine the role of TCLS in vertical femoral neck fracture therapy.
